# Sanitary conditions modulate proinflammatory cytokine response of broilers

**DOI:** 10.1016/j.psj.2025.105984

**Published:** 2025-10-18

**Authors:** Paulina Krzysica, Maarten Hollemans, Aart Lammers, Coen Smits, Huub F.J. Savelkoul, Sonja de Vries, Edwin Tijhaar

**Affiliations:** a Cell Biology and Immunology Group, Wageningen University & Research, Wageningen, the Netherlands; b De Heus, Animal Nutrition, Ede, the Netherlands; c Adaptation Physiology Group, Wageningen University & Research, Wageningen, the Netherlands; d Animal Nutrition Group, Wageningen University & Research, Wageningen, the Netherlands; e Research and Development, Trouw Nutrition, Amersfoort, the Netherlands

**Keywords:** Immunomodulation, Chicken, Sanitary condition, Cytokine, Immune response

## Abstract

Sanitary conditions influence the animal’s adaptive immune response and body weight. Low sanitary conditions (**LSC**), defined as the conditions with high (bacterial) antigenic pressure, were shown to reduce body weight gain of pigs and chickens and increase their natural antibody levels in plasma or serum. The aim of this study was to investigate how sanitary conditions can modulate the inflammatory immune response of broilers. To test this, broiler type chickens kept under LSC or high sanitary conditions (**HSC**) received a respiratory challenge with lipopolysaccharides (**LPS**), and their in vivo and ex vivo immune responses were compared. We measured cytokine levels in plasma before and after LPS challenge, and in culture supernatants of isolated monocytes after the LPS challenge. We found that broilers kept under LSC, in comparison to HSC, had higher systemic interferon (**IFN**)-γ levels in plasma from 0 h to 24 h, and lower IL-10 levels at 48 h after LPS challenge. Monocytes from broilers kept under LSC produced more IL-10 and less IL-12p40 than monocytes from broilers kept under HSC, which resulted in higher IL-10:IL-12 ratios in those chickens. In conclusion, LSC modulated the immune response by inducing a stronger anti-inflammatory (IL-10) response towards the LPS challenge. The exact mechanism behind this modulation still needs to be established, as well as possible consequences of this stronger anti-inflammatory responses for the chicken’s susceptibility to future infections.

## Introduction

Sanitary conditions on farms can affect, among other things, the immune system, and the exposure to less hygienic conditions and higher antigenic pressure influences the subclinical health status of animals (van der Meer et al., 2016). Sanitary conditions differ among applied husbandry systems, such as deep-litter and clean-out systems, but also within these systems, due to variations in their implementations and management (Wilcox et al., 2024). A study on the impact of endotoxins on immune markers showed that chickens on farms with high levels of endotoxins in dust had lower levels of interferon (**IFN**)-γ produced by peripheral blood mononuclear cells (**PBMCs**), a lower proportion of peripheral B cells, and higher corticosterone levels in plasma, suggesting that these animals can be susceptible to pathogenic infections (Roque et al., 2015). Studies in pigs (van der Meer et al., 2016, 2020) tested the impact of low (**LSC**) and high sanitary conditions (**HSC**) on body growth and the immune system. Low sanitary conditions were induced by spreading a mixture of fresh manure and not applying any specific hygiene protocols. It was observed that pigs kept under LSC, in comparison to HSC, had lower body weight and average daily growth (van der Meer et al., 2016, 2020), and higher levels of antigen specific natural antibodies, which are antibodies formed spontaneously without intentional exposure to the corresponding specific antigen (van der Meer et al., 2016). The effect of sanitary conditions on oral tolerance (immune unresponsiveness to ingested antigens) and antibody responses in plasma or serum in broiler type chickens was investigated by Hollemans et al. (2020, 2021), who induced LSC similarly by spreading used litter and not applying specific hygiene protocols. Body weight of broilers kept under LSC was severely reduced (on average 23 % reduction in BW at day 33) in comparison to HSC (Hollemans et al., 2020). Moreover, broilers kept under LSC showed elevated levels of natural antibodies (Hollemans et al., 2020, 2021) and specific antibodies against LPS (IgY) (Hollemans et al., 2021), rabbit γ-globulin (IgM) and bovine serum albumin (IgM and IgY) (Hollemans et al., 2020). These data indicate that LSC modulate antibody responses, which may reflect the higher antigenic pressure and may consequently affect resistance to later infections.

Due to the spreading of used litter, chickens are exposed to high levels of bacterial cell components, such as lipopolysaccharides (**LPS**). Lipopolysaccharide is an endotoxin that is present in the cell wall of gram-negative bacteria and acts as a potent stimulus of the immune system. This pathogen-associated molecular pattern is recognized by Toll-like receptor 4, which is present on a variety of immune cells (Iqbal et al., 2005; Poltorak et al., 1998). The interaction between LPS and Toll-like receptor 4 leads to activation of innate immune cells (Kogut et al., 2005; Peng et al., 2020), such as monocytes and macrophages, resulting in the production of pro-inflammatory cytokines (Takashiba et al., 1999). In in vivo studies, chickens that were introduced to LPS via intratracheal (**i.t.**) treatment developed pulmonary hypertension (Lorenzoni and Wideman, 2008) and initiated primary and secondary antibody responses towards LPS (Parmentier et al., 2006, 2008). Studies on sanitary conditions have mainly focused on animals’ humoral responses but not on cellular responses, such as cytokine release.

The aim of this study was to investigate how sanitary conditions modulate the inflammatory response of broilers by assessing the in vivo and ex vivo cytokine response after a respiratory challenge with LPS. This animal study is a follow up of the experiment described by Hollemans et al. (2020, 2021), where broilers were kept under HSC or LSC to induce subclinical health status. In the current study, we report also an intratracheal challenge with LPS, which is used as a model stimulus to induce an innate immune response. We demonstrate that after LPS challenge, monocytes from broilers kept under LSC produced more IL-10 and less IL-12p40 than from broilers kept under HSC and discuss what this anti-inflammatory phenotype could mean for the broilers ability to cope with future challenges of their immune system.

## Materials and methods

### Ethical approval

All procedures on animals were approved by the Animal Welfare Body of Wageningen University & Research and the Dutch Central Committee on animal experiments (CCD) in accordance with Dutch laws and regulations on the execution of animal experiments no. AVD1040020173026 and no. AVD104002016441.

### Experimental design, animal husbandry, and sanitary conditions

Before the start of the animal experiment, we measured the cytokine production upon LPS stimulation in PBMCs obtained from laying type chickens to determine the period to assess cytokine levels in plasma after i.t. stimulation with LPS in vivo. Based on these insights, an animal experiment was performed, where broiler type chickens were kept under LSC or HSC. Before (0 h) and after (6 h, 24 h, 48 h) i.t. LPS challenge, plasma samples were collected to measure cytokine concentrations. At 48 h post i.t. LPS challenge, heparinized blood was collected from the wing vein to measure cytokine production by ex vivo stimulated monocytes.

The animal experiment was designed as a 2 × 2 factorial approach with early vs delayed feeding strategies and low vs high sanitary conditions as factors (described fully also with vaccine schedule and nutrition in Hollemans et al. (2020)). However, since minimal interactions were present between those factors (Hollemans et al., 2020, 2021), only sanitary conditions were taken into account in this study. Ross 308 male broilers (n = 160 per batch, two consecutive batches) were obtained at 1-day of age and kept in climate respiration chambers (**CRC**) under LSC or HSC. For each batch, one LSC CRC and one HSC CRC was used. Each CRC contained 8 floor pens, and each floor pen accommodated 10 broilers at the start of the experiment. The LSC CRC was under-pressurized (-65 ± 5 Pa), the HSC CRC was over-pressurized (100 ± 5 Pa), and people in contact with animals kept under HSC were obliged to follow a strict hygiene protocol (Hollemans et al., 2020). Both CRC were the same in their set-up and were controlled for the same climate conditions (temperature, humidity, CO2, NH3). In LSC CRC, the sanitary conditions were induced by spreading used-litter that was obtained from commercial broiler farms every 4 days, starting at 3 days of age. At 33 day-of-age of broilers, the LPS challenge experiment started and lasted for 48 h.

Thirty-three-day old broilers were challenged intratracheally with LPS (#L2880, Sigma-Aldrich, Saint Louis, MO) (2.5 mg/kg bodyweight dissolved in 0.5 mL PBS). Blood was collected from one randomly selected chicken per pen (n = 8 per sanitary condition) in heparinized tubes from a wing vein right before LPS inoculation (naive; 0 h) and after the inoculation at 6 h and 24 h time-points for plasma isolation, and at 48 h for plasma and monocyte-derived macrophages isolation and stimulation. Cytokine production of ex vivo stimulated monocytes was only analyzed for one batch of animals, due to a mistake in the LPS-dosing in the first batch.

### Plasma samples

For collection of plasma, the blood was kept in heparinized tubes at RT for 2 h, and then centrifuged at 14,000 g for 10 min. Obtained plasma was aliquoted and stored at −80°C.

### Isolation and Ex Vivo Stimulation of Monocytes

Collected blood was first used to isolate PBMCs as described elsewhere (Verwoolde et al., 2020). Obtained PBMCs were seeded at 1 × 10^6^ cells per well in a 96-well flat bottom plate (#655180, CELLSTAR, Greiner Bio-One, Alphen aan den Rijn, The Netherlands) in 100 µL culture medium and incubated overnight at 41°C, 5 % CO_2_. Subsequently, plates were washed to discard non-adherent cells, while mononuclear adherent cells, mainly monocytes (Verwoolde et al., 2020), remained in the wells. These adherent cells were stimulated with 10 µg/mL *Escherichia coli* LPS (#L2880, Sigma-Aldrich) or medium only (**CTRL**; negative control) and incubated at 41°C, 5 % CO_2_ for 48 h. Cell culture supernatant was collected and stored at −80°C until testing.

### Isolation of PBMCs for in vitro stimulation

Blood from four 16-week-old commercial purebred White Leghorn chickens (Hendriks Genetics, Boxmeer, The Netherlands) was collected from the left or right (randomly selected) wing vein in heparinized tubes at room temperature (**RT**) and processed immediately. The blood was diluted with an equal volume of sterile phosphate-buffered saline (**PBS**; #10010-015, Gibco, Invitrogen, Paisley, UK), placed on top of Histopaque-1.1191 (#1119, Sigma-Aldrich), and centrifuged at 700 g for 40 min at RT. The interface was collected and washed 3 x with sterile PBS at 250 g for 8 min at RT. The cell pellet was resuspended in complete culture medium Roswell Park Memorial Institute (**RPMI 1640**; #52400-025, Gibco, Invitrogen) with the addition of 10 % (v/v) heat-inactivated chicken serum (#16110082, Gibco, Invitrogen), 100 units/mL penicillin and 100 µg/mL streptomycin (#15140122, Gibco, Invitrogen), and 2 mM l-glutamine (#25030024, Gibco, Invitrogen).

### Stimulation of PBMCs

The PBMCs stimulation was performed to study the cell responsiveness and cytokine production to LPS stimulation. The isolated PBMCs were counted in a Bürker chamber using trypan blue exclusion to count life cells, and seeded in a 96-well flat bottom plate (3596, Corning Inc., Corning, NY) at 0.5 × 10^6^ cells per well in 100 µL. Cells were stimulated by the addition of 100 µL E.coli LPS or Pokeweed Mitogen (**PWM**) (#L9379, Sigma-Aldrich), resulting in final concentrations of 10 µg/mL LPS or 20 µg/mL PWM. Non-stimulated cells were included as a negative control. All conditions were performed in triplicate for PBMCs from four chickens. Cells were incubated at 41°C, 5 % CO_2_, and cell culture supernatant was collected after 4 h, 10 h, 24 h, and 48 h of incubation. Right before the harvesting, the plates were centrifuged at 350 g for 4 min. The supernatant was then stored at −80°C.

### Capture ELISA

The full protocol for the capture ELISAs was described elsewhere (Krzysica et al., 2022). In short, ELISA high-binding, 96-well, flat-bottom plates (#655061, Greiner Bio-One, Kremsmünster, Austria) were coated with capture antibody and incubated overnight. Subsequently, the plates were blocked for 1 h and washed with washing solution. Plasma and PBMCs supernatant samples (with 0.5 % heat-inactivated normal rabbit serum) or monocytes supernatant samples (without rabbit serum) were diluted in 1.5 % (w/v) bovine serum albumin in PBS and were added to the plates and incubated as described previously. The plates were washed, and the biotinylated detection antibody was added and incubated for 1 h. The plates were washed again, and streptavidin (Streptavidin poly-HRP80, #SP80C, SDT GmbH, Kraichtal, Germany) solution was added and incubated for 30 min. After incubation, the plates were washed, and enhanced K-Blue TMB Substrate (#308177, Neogen, Lexington, KY) was added to the plates. Color development was stopped by adding the stop buffer (0.65 M HCl in demi water). The plates were measured by FilterMax F5 spectrophotometer (Molecular Devices, San Jose, CA) at 450 nm (OD_450_), from which the OD_620_ was subtracted. The cytokine concentrations were calculated using a five-parameter logistic curve fit in the program Soft-Max Pro v.6.2.2 and v.7.1.0 (Molecular Devices, San Jose, CA). Unfortunately, IFN-γ from PBMCs supernatant had missing values due to accidental loss of two samples, therefore IFN-γ data from only two chickens were reported.

### Statistical analyses

Data were processed and analyzed using R version 3.6.1 (R Development Core Team). Effects of in vitro LPS and PWM stimulation on cytokine production by PMBCs was evaluated within incubation time (4, 10, 24, 48 h) by ANOVA with stimulation (no, LPS, PWM) as fixed effect. Cytokine levels from plasma before and after LPS challenge in chickens kept under LSC vs HSC were evaluated within time point using a Kruskall-Wallis test. Cytokine production of ex vivo stimulated monocytes form broilers kept under LSC or HSC after LPS challenge were evaluated by ANOVA with stimulation (no, LPS) or sanitary conditions (high, low) as fixed effects. Cytokine concentrations were log-transformed prior to analyses.

The ratio between IL-10:IL-12 production by ex vivo stimulated monocytes form broilers kept under LSC or HSC after LPS challenge were evaluated by a two-tailed Mann-Whitney test with 95 % confidence level using GraphPad Prism 9.3.1 (GraphPad Software, San Diego, California).

Model residuals of the linear models were visually assessed by QQ-plots and residual plots to verify assumptions of normality and homogeneity. Differences among means were tested using type III least squares statistics. P-values ≤ 0.05 were considered statistically significant and P-values ≤ 0.10 were considered as tendencies. Data are presented as box-whiskers plot of raw values.

## Results

### Cytokine production after stimulation of PBMCs with LPS and PWM

Prior to the actual in vivo study, a preliminary experiment was performed where the cytokine response of White Leghorn PBMCs to in vitro stimulation with LPS was assessed to indicate when to measure cytokine levels in plasma after in vivo (intratracheal) stimulation with LPS. We measured post stimulation protein levels of chicken cytokines IL-2, IL-6, IL-10, IL-12p40, and IFN-γ in the supernatant of stimulated PBMCs at different timepoints, using our recently developed capture ELISAs (Krzysica et al., 2022). Pokeweed mitogen was used as a positive control, because this mitogen is known to be a potent cytokine inducer in mammalian PBMCs (Katial et al., 1998), as well as in chicken PBMCs (Krzysica et al., 2022).

**Fig. 1:**
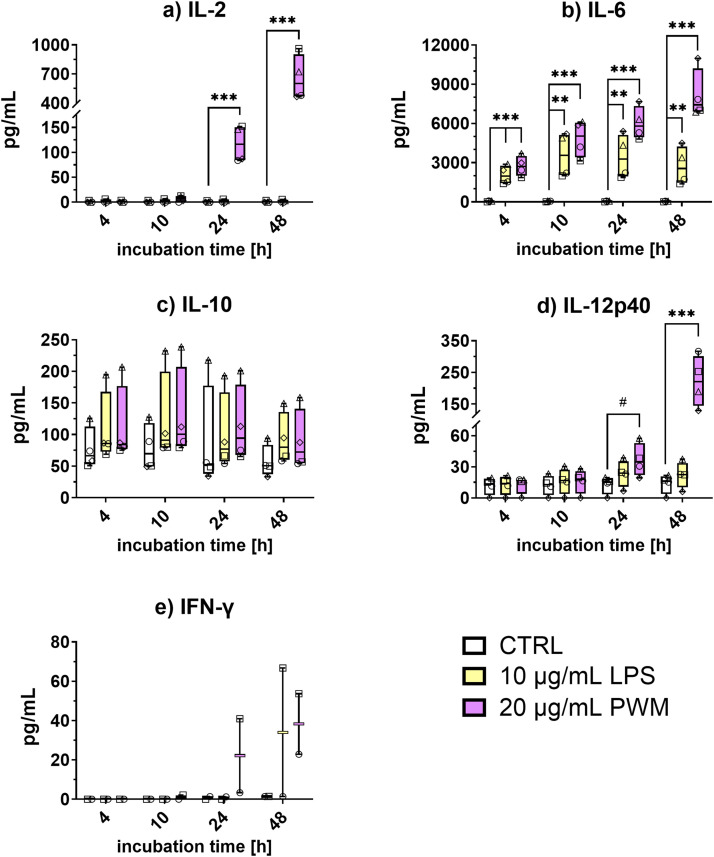
Production of cytokines upon in vitro mitogenic stimulation of White Leghorn peripheral blood mononuclear cells (PBMCs). PBMCs were stimulated with lipopolysaccharides (LPS; 10 µg/mL), pokeweed mitogen (PWM; 20 µg/mL) or not-stimulated (CTRL; negative control) for 4 h, 10 h, 24 h or 48 h. Cell culture supernatant was collected and analysed by capture ELISA for the production of a) IL-2, b) IL-6, c) IL-10, d) IL-12p40 (n = 4) and e) interferon (IFN)-γ (n = 2). Data points for each individual animal are represented with a separate symbol. The data are presented as boxes extending from the 25th to 75th percentiles with horizontal black line indicating median. Whiskers indicate minimum and maximum values, and symbols indicate individual observations. Differences among groups are indicated with: # P < 0.1; * P < 0.05; ** P < 0.01; or *** P < 0.001.

Stimulation with PWM induced IL-2, IL-6, IL-12p40, and IFN-γ, and the mean concentrations of these cytokines reached maximum levels in the culture supernatant at 48 h. In particular, IL-2 and IL-12p40 were produced at higher levels when compared to the non-stimulated (CTRL) condition (IL-2: 766-fold, P < 0.001; IL-12p40: 16-fold, P < 0.001) (Fig. 1). Interferon-γ levels started increasing from 24 h (32-fold) and were the strongest at 48 h (27-fold). Interleukin-6 could already be detected in the supernatant at 4 h (61-fold, P < 0.001), and also reached maximum levels at 48 h (214-fold, P < 0.001) post PWM stimulation. Stimulation with LPS induced IL-6 at all assessed time-points from 4 h to 48 h, ranging from around 2000 pg/mL at 4 h (46-fold, P < 0.001) to a peak value around 3500 pg/mL at 10 h (84-fold, P = 0.003). Lipopolysaccharides, similarly to PWM, also induced maximal level of IFN-γ at 48 h (24-fold). However, production of IFN-γ could only be assessed for two animals. This makes it impossible to statistically test these results, that are therefore presented descriptively. Stimulation with either mitogen resulted in IL-10 levels that were not higher compared to the unstimulated control (P > 0.601). Moreover, measured concentrations of IL-10 were constantly higher for one (triangle) of the four tested animals. Since this animal (triangle) showed the same IL-10 production profile as the other animals, except at a considerably higher level, it was considered to be a high responder rather than an outlier. Overall, these preliminary results showed that the highest cytokine responses after in vitro mitogenic stimulation of PBMCs were observed at 48 hours post stimulation.

### Sanitary conditions influence the plasma levels of IFN-γ and IL-10

**Fig. 2:**
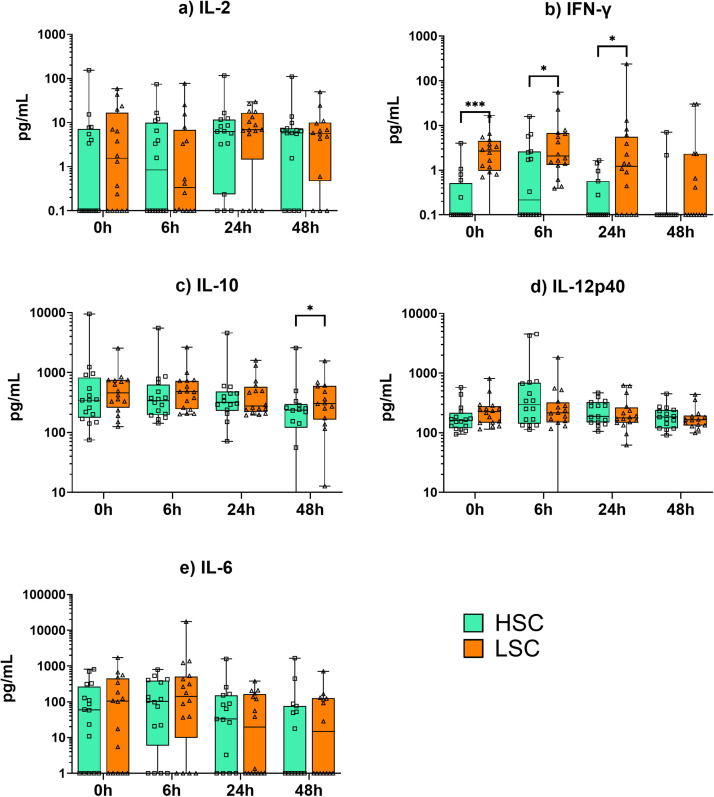
Concentration of cytokines in plasma of broiler chickens kept under high and low sanitary conditions (HSC and LSC) upon an intratracheal lipopolysaccharides (LPS) challenge. Broilers kept under HSC (n = 16) and LSC (n = 16) were intratracheally challenged with 2.5 mg LPS per kg bodyweight. Plasma samples were collected immediately before treatment (0 h) or at 6 h, 24 h, 48 h after treatment. Cytokines a) IL-2, b) interferon (IFN)-γ, c) IL-10, d) IL-12p40, and e) IL-6 were measured. The data are presented as boxes extending from the 25th to 75th percentiles with horizontal black line indicating median. Whiskers indicate minimum and maximum values, and symbols indicate individual observations. Differences among groups are indicated with: * P < 0.05; ** P < 0.01; or *** P < 0.001.

The impact of sanitary conditions on the inflammatory response was assessed by measuring cytokine levels in plasma after intratracheal challenge with LPS (Fig. 2). Because mitogenic in vitro stimulation of isolated chicken PBMCs showed the most convincing cytokine responses after 48 hours (paragraph 3.1), cytokine levels were assessed for a period of 48 hours following intratracheal LPS challenge. Immediately before LPS administration (0 h), no differences in any of the cytokine levels were observed between broilers housed at different sanitary conditions, except for IFN-γ (Fig. 2). This cytokine was produced at higher levels in broilers kept under LSC, compared to HSC, already at 0 h (8-fold, P < 0.001). After administration of LPS, IFN-γ levels were still higher at 6 h (3-fold, P = 0.023) and 24 h (57-fold, P = 0.017) for LSC than HSC broilers, but these differences disappeared at 48 h (8-fold, P = 0.086). At 48 h post LPS administration, higher levels of IL-10 were observed in broilers kept under LSC compared to broilers kept under HSC (1.1-fold, P = 0.048). No effects of sanitary conditions on the cytokine levels of IL-2, IL-6, and IL-12p40 were observed. To summarize, higher plasma levels of IFN-γ were observed in broilers kept under LSC than under HSC, before (0 h) and in the first 24 hours after LPS challenge. Higher plasma levels of IL-10 were observed only at 48 h in the broilers kept under LSC compared to the HSC.

### Ex vivo LPS-stimulated monocytes from broilers kept under LSC produce reduced levels of IL-12p40 and elevated levels of IL-10

**Fig. 3:**
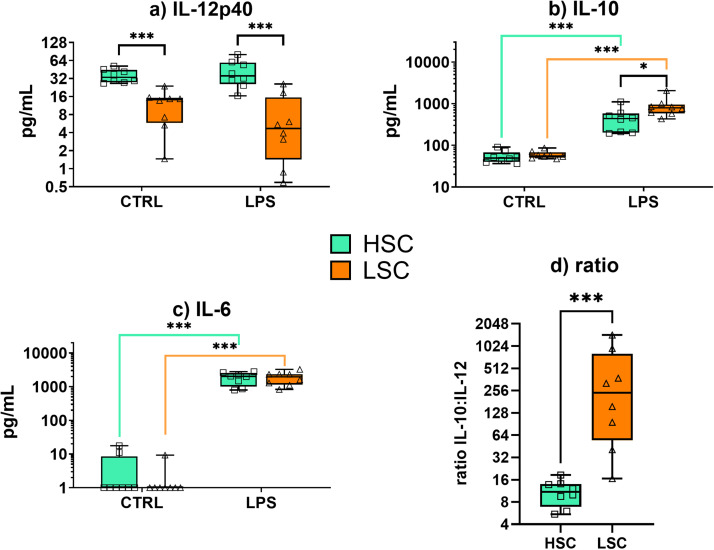
Cytokine production upon lipopolysaccharides (LPS) stimulation of primary monocytes from broiler chickens kept under high and low sanitary conditions (HSC and LSC). Monocytes were stimulated with 10 µg/mL LPS or were not-stimulated (CTRL; negative control) for 48 h. Cell culture supernatant was tested by capture ELISA for production of a) IL-12p40, b) IL-10 and c) IL-6 in broilers from HSC (n = 8) and LSC (n = 8). d) Cytokines IL-10 and IL-12p40 are presented as ratio of IL-10:IL-12p40 from monocytes stimulated with LPS. The data are presented as boxes extending from the 25th to 75th percentiles with horizontal black line indicating median. Whiskers indicate minimum and maximum values, and symbols indicate individual observations. Differences among groups are indicated with: * P < 0.05; ** P < 0.01; or *** P < 0.001.

After measuring the cytokine response in plasma for 48 hours, chickens were sacrificed and monocytes were isolated and stimulated with LPS to further assess if the innate immune system was modified by the sanitary housing conditions (Fig. 3). Ex vivo stimulation with LPS did not result in enhanced production of IL-12p40 compared to unstimulated (CTRL) cells (LSC: P = 0.086; HSC: P = 0.967). However, when comparing sanitary conditions, the IL-12p40 production by unstimulated (CTRL) monocytes from broilers kept under LSC was 3-fold lower than for unstimulated monocytes from broilers kept under HSC (P < 0.001). After LPS stimulation, monocytes from broilers kept under LSC produced even 5-fold lower IL-12p40 levels than the HSC monocytes (P < 0.001) (Fig. 3a).

IL-10 was induced by ex vivo LPS stimulation in broilers from both sanitary conditions (LSC: 15-fold, P < 0.001; HSC: 8-fold, P < 0.001) (Fig. 3b). Moreover, in LPS-stimulated monocytes, the cells from broilers kept under LSC produced 2-fold higher levels of IL-10 compared to the HSC (P = 0.02). Stimulation with LPS resulted in increased production of IL-6 compared to the CTRL (LSC: 1600-fold, P < 0.001; HSC: 500-fold, P < 0.001), but no differences between the monocytes from broilers kept under LSC and HSC were observed (CTRL: P = 0.383; LPS: P = 0.937) (Fig. 3c). Overall, LSC induced lower IL-12p40 and higher IL-10 levels compared to HSC, and IL-6 did not differ between the groups.

Next, the ratio between production of the anti-inflammatory cytokine IL-10 and the pro-inflammatory cytokine IL-12p40 was calculated (Fig. 3d). The mean ratio was 425 for monocytes from broilers kept under LSC and 11 for HSC. Thus, after intratracheal LPS challenge, the anti-inflammatory IL10:IL-12p40 ratio was nearly 40-fold higher after ex vivo LPS stimulation in the LSC monocytes than in the HSC monocytes (P < 0.001).

## Discussion

**Fig. 4:**
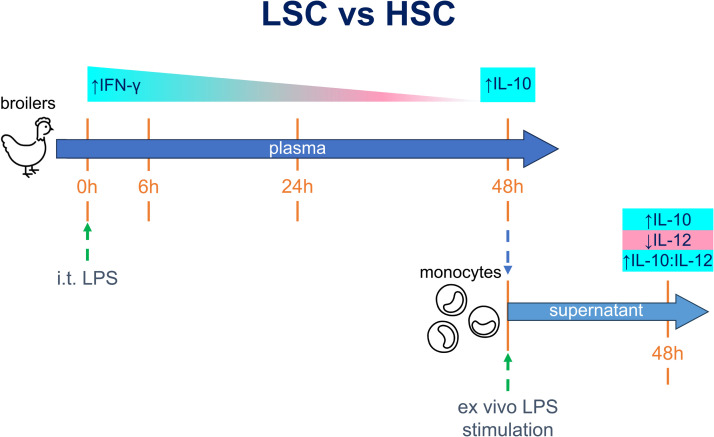
Overview of the results from in vivo and ex vivo experiments in relation of low sanitary conditions (LSC) to high sanitary conditions (HSC). Broilers’ plasma was collected before (0 h) and after (6 h, 24 h, and 48 h) intratracheal (i.t) LPS challenge. At 48 h, monocytes were isolated from the blood and stimulated with LPS for 48 h. Arrows beside cytokines indicate the relative levels in broilers kept under LSC compared to HSC.

Various housing stressors have an impact on the immune system of poultry (Hofmann et al., 2020), including sanitary conditions (Hollemans et al., 2020, 2021; Roque et al., 2015). The aim of this study was to investigate if sanitary conditions can influence broilers’ inflammatory responses, using a respiratory LPS challenge model. Lipopolysaccharides are abundantly present in the chicken environment (Roque et al., 2015) and used as a model stimulus to induce an innate immune response. In the current study, we compared the effects of LSC and HSC on the innate response after a pro-inflammatory challenge with LPS. The main findings of this study are summarized in Fig. 4. We observed higher plasma levels of IFN-γ before LPS challenge (0 h) and up to 24 hours after the challenge in broilers kept under LSC compared with HSC. However, the difference in IFN-γ levels between the broiler kept under LSC and HSC gradually decreased, until it was lost at 48 h. Initially, no differences were observed in IL-10, until 48 h, at which timepoint the broilers kept under LSC produced more IL-10. Furthermore, the isolated monocytes from broilers kept under LSC stimulated ex vivo with LPS (Fig. 4) produced lower levels of IL-12p40, higher levels of IL-10, and had higher anti-inflammatory IL-10:IL-12 ratio than monocytes from broilers kept under HSC.

The cytokines that differed most between broilers kept under LSC and HSC were in vivo IFN-γ and IL-10, and ex vivo IL-10 and IL-12p40. Interferon-γ is a signature cytokine for T helper-1 (**Th1**) cells (Schroder et al., 2004), also in chickens (Degen et al., 2004; Mallick et al., 2011). These Th1 cells play a crucial role in the immune system, particularly in fighting off pathogens like viruses and intra- and extracellular bacteria (Berger, 2000; Mosmann and Moore, 1991). The higher levels of IFN-γ in plasma of broilers kept under LSC indicate the presence of higher numbers of activated Th1 cells, likely due to the higher exposure to microbes under these LSC. Despite the fact that IFN-γ was higher in broilers kept under LSC than HSC for the first 24 h after LPS challenge in vivo, IL-10 production was higher in broilers kept under LSC in vivo 48 h post challenge and ex vivo upon LPS re-stimulation. Interleukin-10 is an anti-inflammatory cytokine that plays an important role in controlling inflammatory reactions. It can repress the production of inflammatory cytokines like TNF-α and IFN-γ (Hu et al., 2006; Moore et al., 2001; Rothwell et al., 2004; Schroder et al., 2004). The relatively higher in vivo IL-10 levels 48 h after i.t. LPS challenge in broilers kept under LSC versus HSC may have contributed to the reduced IFN-γ levels at 48 h in LSC broilers compared to 0 h. Monocytes from broilers kept under LSC produced after 48 h ex vivo stimulation with LPS considerably less IL-12p40 than monocytes of broilers kept under HSC. Interleukin-12 is mostly produced by antigen presenting cells like dendritic cells and macrophages/monocytes. It is an inflammatory cytokine that plays an important role in the initiation of adaptive immune responses to viruses and bacteria (Hu et al., 2006; Rothwell et al., 2004; Schroder et al., 2004). The reduced inflammatory IL-12 levels and increased anti-inflammatory IL-10 levels in LPS-stimulated ex vivo monocytes of broilers kept under LSC resulted in a markedly increased anti-inflammatory ratio of IL-10:IL-12 in these broilers. Interleukin-10 acts as a feedback regulator of these Th1 immune responses by suppressing IL-12 production, while at the same time controlling inflammatory responses during bacterial infections (Hu et al., 2006; Rothwell et al., 2004; Schroder et al., 2004). Taken together, these results indicate higher activity of IFN-γ-producing Th1 cells in broilers kept under LSC, while their (innate) immune system may have shifted to a more anti-inflammatory modus that aims to avoid excessive immune mediated damage, as indicted by the reduced IL-12p40 and higher IL-10 production by isolated monocytes.

The higher IFN-γ level in plasma of broilers kept under LSC might at first sight appear in contrast to the study of Roque et al. (2015). That study showed lower IFN-γ production by concanavalin A-stimulated PBMCs from chickens kept at farms with higher endotoxin concentration in dust (uncontrolled equivalent of LSC) than PBMCs of chickens kept at farms with lower endotoxin concentration in dust (uncontrolled equivalent of HSC). However, the higher IFN-γ plasma levels observed in broilers kept under LSC in our study was likely caused by more activated antigen specific Th1 cells in broilers kept under LSC as a result of increased exposure to microbes. In the study of Roque et al. (2015), IFN-γ was measured in PBMCs stimulated ex vivo with concanavalin A, a mitogenic stimulus that activates all T cell, irrespective of antigen presence (Hovi et al., 1978). The lower level of concanavalin A-induced IFN-γ by PBMCs from chickens housed at high endotoxin levels, in comparison with chickens housed at low endotoxin levels, is actually in line with the more anti-inflammatory phenotype observed in our study in re-stimulated monocytes from broilers kept under LSC.

Apart from this anti-inflammatory phenotype observed in monocytes, other mechanisms, such as regulatory T cells (**Treg**) activity or LPS tolerance, could in theory also explain the enhanced anti-inflammatory phenotype of broilers kept under LSC. Regulatory T cells could possibly influence the switch in the immune responses through their regulatory role, helping to avoid harmful consequences. We observed higher plasma levels of IL-10 in broilers kept under LSC than in HSC at 48 h after LPS challenge (Fig. 2). Although IL-10 can also be produced by monocytes (Fig. 3), it is possible that this stronger induction of plasma IL-10 was the result of Treg activity to reduce inflammation and immunopathology.

Finally, LPS or endotoxin tolerance, characterized by a temporal decreased LPS response after prolonged exposure to endotoxins (Biswas and Lopez-Collazo, 2009), could also be a possible explanation for the immune response in broilers kept under LSC. Those broilers were subjected to prolonged exposure to high bacterial antigen content (including LPS) due to the spreading of used litter. This may be reflected by the higher plasma levels of IFN-γ, a cytokine mostly produced by activated T and NK cells (Schroder et al., 2004), before intratracheal LPS challenge in these broilers compared to the broilers kept under HSC. After the subsequent i.t. challenge with LPS, the initially higher plasma IFN-γ levels became reduced in these broilers kept under LSC, and showed after 48 hours no significant differences anymore with those kept under HSC. However, a modest but significantly higher IL-10 plasma level was then observed in broilers kept under LSC compared to HSC. It was shown that enhanced expression of IL-10 is part of the mechanism for LPS tolerance (Ziegler-Heitbrock, 1995). The broilers kept under LSC also produced less IL-12p40 ex vivo when compared to HSC. Endotoxin tolerance could be a protective mechanism against the lethal outcome of a septic shock or inflammatory damage induced by cytokines (Seeley and Ghosh, 2017), which would be relevant for broilers kept under LSC that are under constant high load of environmental bacteria and endotoxins. While described in mammals, the induction and underlying molecular mechanisms of LPS tolerance in chickens needs to be elucidated further (De Boever et al., 2008; Verwoolde et al., 2020, 2021; Wideman et al., 2009). Taken together, endotoxin tolerance could be a possible mechanism induced in broilers kept under LSC as adaptation to environment with high bacterial antigen content to limit immune mediated damage.

We speculate that, in comparison to the broilers kept under HSC, the prolonged interactions of broilers kept under LSC with a higher pathogenic load caused higher production of IFN-γ by antigen-specific T cells and by NK cells to deal with this antigenic pressure. Nevertheless, the in vivo challenge with LPS reduced IFN-γ and induced IL-10, suggesting the induction of a negative feedback loop to the induced Th1 response as described above to limit immune mediated collateral damage. Ex vivo stimulation of monocytes from broilers kept under LSC showed higher IL-10 and lower IL12p40 levels compared to HSC, with an anti-inflammatory IL-10:IL-12 ratio that was nearly 40-fold higher for the monocytes from broilers kept under LSC than HSC. We postulate that this shift towards an anti-inflammatory response may be due to tolerance induction by LPS and/or by enhanced Treg activity caused by prolonged exposure to bacterial antigens prior to intratracheal LPS challenge.

In conclusion, LSC appear to skew the immune response in broilers towards a more anti-inflammatory direction, as demonstrated by increased production of IL-10, with a concomitant decreased production of IL-12p40 and IFN-γ. We speculate that this anti-inflammatory shift induced by LSC may reduce immune-mediated inflammation and accompanied tissue damage. Future challenge studies will have to show if this anti-inflammatory skewing by LCS make broilers more susceptible to microbial infections.

## CRediT authorship contribution statement

**Paulina Krzysica:** Writing – review & editing, Writing – original draft, Visualization, Validation, Methodology, Investigation, Formal analysis, Conceptualization. **Maarten Hollemans:** Writing – review & editing, Resources, Methodology, Investigation, Formal analysis, Conceptualization. **Aart Lammers:** Writing – review & editing, Supervision, Project administration, Methodology, Conceptualization. **Coen Smits:** Writing – review & editing, Supervision, Funding acquisition. **Huub F.J. Savelkoul:** Writing – review & editing, Writing – original draft, Supervision, Project administration, Methodology, Funding acquisition, Conceptualization. **Sonja de Vries:** Writing – review & editing, Supervision, Project administration, Methodology, Funding acquisition, Conceptualization. **Edwin Tijhaar:** Writing – review & editing, Writing – original draft, Supervision, Project administration, Methodology, Funding acquisition, Conceptualization.

## Disclosures

The authors declare the following financial interests/personal relationships which may be considered as potential competing interests:

Paulina Krzysica reports financial support was provided by Fund of the European Union and European Regional Development Fund, grant number PROJ 00661, jointly financed by Wageningen University & Research, Trouw Nutrition, Micreos, NYtor, and Agri Information Partners. Maarten Hollemans reports financial support was provided by Coppens Diervoeding B.V, Helmond, the Netherlands. Co-author employed by Coppens Diervoeding B.V (Helmond, the Netherlands), current affiliation De Heus (Ede, the Netherlands) - M.H. Co-author employed by Trouw Nutrition (Amersfoort, the Netherlands) - C.S. If there are other authors, they declare that they have no known competing financial interests or personal relationships that could have appeared to influence the work reported in this paper.
